# Butyrate Metabolism‐Related Gene Signature in Tumor Immune Microenvironment in Lung Adenocarcinoma: A Comprehensive Bioinformatics Study

**DOI:** 10.1002/iid3.70087

**Published:** 2024-12-06

**Authors:** Jing Zhao, Xueyue Wang, Jing Wang, Yating You, Qi Wang, Yuan Xu, Ye Fan

**Affiliations:** ^1^ Department of Clinical Skills Training Center Xinqiao Hospital, Army Medical University Chongqing China; ^2^ Department of Paediatrics General Hospital of Xizang Military Region Xizang China; ^3^ Department of Respiratory Disease Xinqiao Hospital, Army Medical University Chongqing China; ^4^ Department of Preventive Medicine Xinqiao Hospital, Army Medical University Chongqing China; ^5^ Department of Orthopaedics Xinqiao Hospital, Army Medical University Chongqing China

**Keywords:** butyrate, gene signature, LUAD, metabolism, tumor immune microenvironment

## Abstract

**Background:**

Experimental results have verified the suppressive impact of butyrate on tumor formation. Nevertheless, there is a limited understanding of the hidden function of butyrate metabolism within the tumor immune microenvironment (TIME) of lung adenocarcinoma (LUAD). This research aimed at digging the association between genes related to butyrate metabolism (butyrate metabolism‐related genes [BMRGs) and immune infiltrates in LUAD patients.

**Methods:**

Through analyzing The Cancer Genome Atlas dataset (TCGA), the identification of 38 differentially expressed BMRGs was made between LUAD and normal samples. Later, a prognostic signature made up of nine BMRGs was made to evaluate the risk score of LUAD subjects. Notably, high‐risk scores emerged as negative prognostic indicators for overall survival in LUAD subjects. Additionally, BMRGs displayed associations with immunocyte infiltration levels, immune pathway activities, and pivotal prognostic hub BMRGs.

**Results:**

One key prognostic BMRG, PTGDS, exhibited a robust correlation with T cells, the chemokine‐related pathway, and the TCR signaling pathway. This study suggests that investigating the interplay between butyrate metabolism and T cells could present a promising novel approach to cancer treatment. OncoPredict analysis further unveiled distinct sensitivities of nine medicine in high‐ and low‐risk groups, facilitating the selection of optimal treatment strategies for individual LUAD patients.

**Conclusions:**

The study establishes that the BMRG signature serves as a sensitive predictive biomarker, providing profound insights into the crucial effect of butyrate metabolism in the context of LUAD TIME.

## Graphical abstract

We established a novel gene signature associated with butyrate metabolism, serving as a sensitive predictive biomarker for the prognosis of LUAD patients. We underscored the necessity for further comprehensive investigations to unveil the regulatory mechanisms governing butyrate metabolism in LUAD.

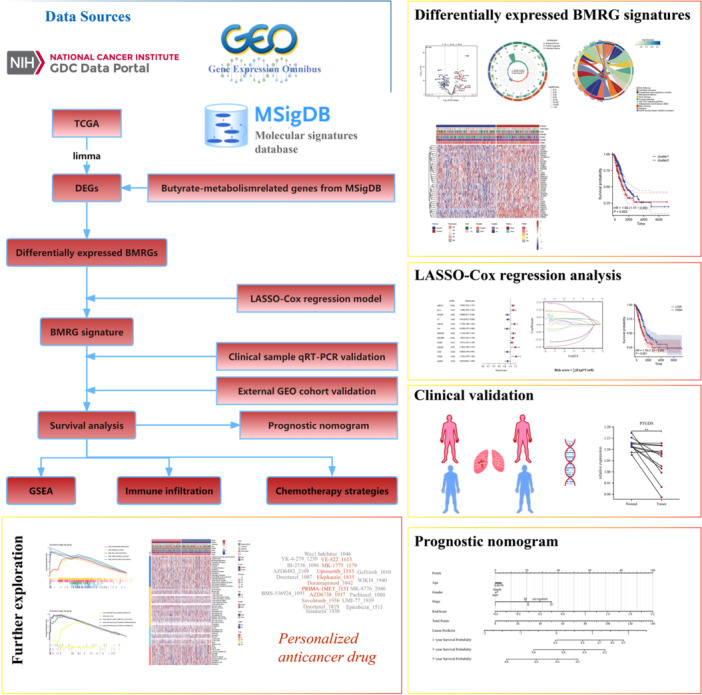

## 1 Introduction

Lung cancer accounts for the main cause of cancer deaths worldwide [1]. Lung adenocarcinoma (LUAD) is the most ordinary histological subtype of non‐small‐cell lung cancer, and the search for potential therapeutic targets continues to intensify [2, 3, 4]. Despite of much advanced progress in the LUAD treatment, the primary challenges in LUAD treatment remain distant metastases and resistance to targeted therapeutics [5]. Therefore, identifying novel therapeutic targets and prognostic markers is crucial for predicting patient survival and guiding clinical management in LUAD patients.

Short‐chain fatty acids, such as butyrate, are produced when anaerobic bacteria in the colon metabolize food fibers [6]. The relationship between human gut microbiota and cancer is becoming more and more clear, whether it be through numerous microbial populations or particular pathogens, particularly their metabolites [7]. As a typical metabolite, butyrate plays an anti‐cancer effect by speeding the death and differentiation of cancer cells as well as inhibiting uncontrolled malignant tendencies and inflammation [8, 9, 10, 11, 12]. By affecting the histone acetylation patterns and changing the expression of genes involved in the cell cycle, including microRNA, butyrate can inhibit the growth of cancer cells [13, 14, 15]. Besides, Broadfield et al. [16] reported that the cancerous risk, progression, and severity are all indicated by a decreasing fecal butyrate level. Furthermore, the latest scientific findings found that butyrate can mediate the differentiation and expression of pro‐inflammatory cytokines and distinct immunocytes in cancer [17, 18]. In immune and cancer cells, intracellular butyrate increases the number of acetylated histones by preventing the activity of the enzyme histone deacetylase (HDAC), which controls the levels of transcription factors and proteins that are engaged in important regulating pathways [19].

While the accumulating evidence mentioned above suggests that butyrate exhibits anticancer effects, there is ongoing research regarding the regulatory impact of butyrate on immune infiltration and the butyrate metabolism landscape within the tumor immune microenvironment (TIME) of LUAD. The complexities of these interactions remain incompletely understood. Therefore, we conducted this study to systematically analyze butyrate metabolism in LUAD, aiming to deeply understand its effect on the disease.

This research adopted bioinformatics technologies to assess the predictive significance of butyrate metabolism in LUAD datasets obtained from the The Cancer Genome Atlas dataset (TCGA) and Gene Expression Omnibus (GEO) databases. A prognostic signature encompassing nine genes strongly associated with LUAD prognosis was identified, enabling the calculation of a butyrate metabolism‐associated risk score. Subsequently, we explored the impact of risk marks on immunocyte infiltration and immune‐related pathways, revealing a great influence of butyrate metabolism on the LUAD TIME. Furthermore, a nomogram was developed to forecast the prognosis of LUAD patients with diverse clinical features. Finally, we evaluated the chemotherapeutic drug sensitivity of different risk groups.

## 2 Materials and Methods

### 2.1 Data Collection and Preprocessing

**Figure 1:**
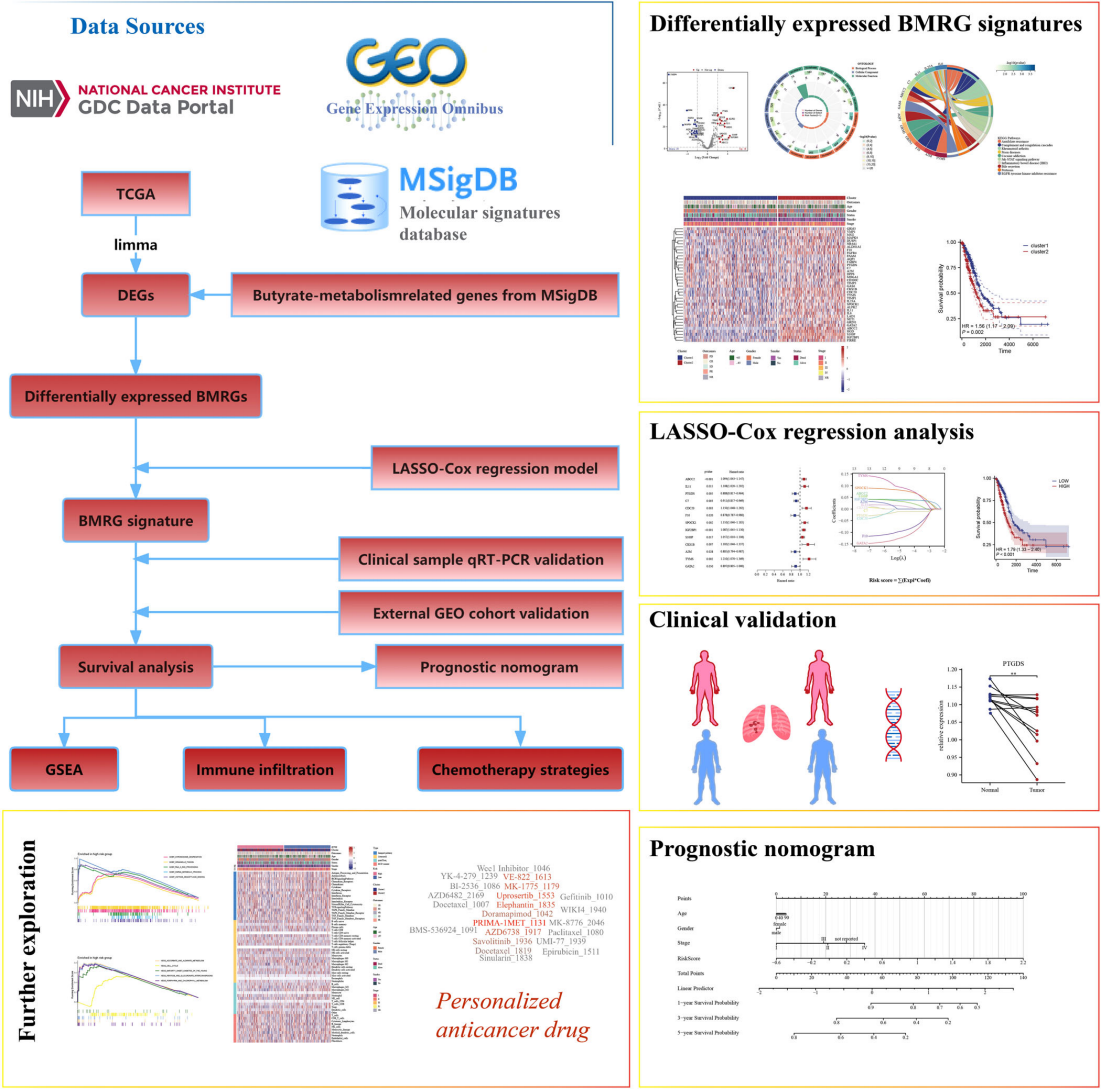
The flowchart of the study.

The flowchart of the study was shown in Figure 1. The clinical information and RNA‐sequencing information of the LUAD patients were acquired from the TCGA and GEO databases. Before analysis, all the transcriptome's fragments per kilobase million information were log‐transformed to transcripts per million. A total of 513 LUAD patients from the TCGA made up the training cohort, while 739 LUAD patients from three eligible LUAD cohorts (GSE72094 [20], GSE31210 [21], and GSE26939 [22]) in the GEO database made up the validation cohort. Besides, the 348 butyrate‐metabolism‐associated genes (Supporting Information: Table S1) were provided by the Gene Set Enrichment Analysis database (https://www.gsea-msigdb.org/gsea/index.jsp), and researchers can input “butyrate” in the Human Collections to retrieve these genes, the similar strategy was reported in other study [23].

### 2.2 Identification of Butyrate‐Metabolism‐Related Differentially Expressed Genes (DEG)

The R package “limma” was adopted to recognize the DEGs between LUAD samples and normal samples in the TCGA cohort, and the modified *p* < 0.05 and absolute log2 fold change >1.5 were determined as the cut‐off criteria [24]. Then, by taking the intersection of the DEGs and the butyrate metabolism‐associated genes (butyrate metabolism‐related genes [BMRGs]), the identification of differentially expressed BMRGs was made. The main component analysis was next made utilizing the R package “factoextra” to examine the discrimination ability of these gene‐expression modes between LUAD and normal tissues. Afterwards, the expression pattern, GO and KEGG function of these BMRGs were analyzed. Besides, the genetic mutation data for LUAD were provided by the TCGA dataset, and the R package “maftools” was adopted to visualize the mutation information of selected genes.

### 2.3 Unsupervised Consensus Clustering of BMRGs in LUAD Samples

We utilized the R package “ConsensusClusterPlus” to classify the subgroups of LUAD patients on basis of BMRGs expression levels to examine the prognostic impact of various butyrate metabolism patterns [25]. The partition around medoids (PAM) algorithm was employed, and to regulate the robustness of the clustering, 1000 iterations were carried out with every iteration containing 80% of the samples [26, 27]. The ideal cluster number was established using the consensus score's cumulative distribution function (CDF) curve. The survival curves for every cluster was assessed with the Kaplan–Meier approaches.

### 2.4 Construction and Validation of a Prognostic Signature Based on Hub BMRGs

First, univariate Cox regression via the R package “survival” was adopted to screen out prognosis‐associated genes in the TCGA training set. The candidate genes were then further reduced with the least absolute shrinkage and selection operator regression applying the R package “glmnet,” and the prognostic signature was established.

The risk mark calculating formula is shown below:

### 2.5 Risk Mark = ∑(Expi × Coefi)

The terms risk parameter and gene expression, are denoted by Coefi and Expi. Patients from the training and verification cohorts were isolated into low‐risk and high‐risk categories on basis of the average risk mark of the training cohort.

Subsequently, the prognostic value of the clinical model was assessed with time‐dependent receiver operating characteristic curves and Kaplan–Meier survival curves, and the R packages “survminer” and “survival” and “pROC” implemented the above processes. In addition, three independent datasets (GSE72094, GSE31210, and GSE26939) from GEO database were utilized for external verification to confirm the robustness of the butyrate‐metabolism signature.

### 2.6 Prognostic Signature Model‐Based Subgroup Analysis and Clinical Characteristic Comparison

We performed subgroup analysis based on age, sex, and pathological TNM stages to further analyze the predictive significance of the signature model (Table 1). With the use of the R package “survival,” prognostic markers independently related to the total survival of LUAD patients were recognized by performing univariate and multivariate Cox regression analysis. Afterwards, with the R package “rms,” a predictive nomogram comprised of risk, gender, stage, and age was established [28]. Based on the matching scores for each variable, the total score for each subject was determined. The predictive power between the anticipated 1–5‐year overall survival (OS) rates and the actual results obtained was evaluated using the calibration plot. To assess the precision of the nomogram's forecast of 1–5‐year OS, time‐dependent AUC values were calculated.

**Table 1:** Univariate and multivariate Cox regression analysis results for prognostic markers in LUAD patients.

		Univariate analysis		Multivariate analysis	
Characteristic	Total (*N*)	Hazard ratio (95% CI)	*p* value	Hazard ratio (95% CI)	*p* value
Age	513	0.999 (0.989–1.009)	0.855		
Gender	513		0.573		
Female	276	Reference			
Male	237	1.087 (0.814–1.452)	0.573		
Stage	513		<0.001		
I	280	Reference		Reference	
II	120	2.016 (1.431–2.840)	<0.001	1.906 (1.352–2.688)	<0.001
III	80	2.006 (1.342–2.998)	<0.001	1.831 (1.223–2.740)	<0.01
IV	25	3.050 (1.680–5.537)	<0.001	3.044 (1.674–5.537)	<0.001
Not reported	8	2.030 (0.638–6.458)	0.230	2.417 (0.757–7.712)	0.136
RiskScore	513	3.153 (2.258–4.403)	<0.001	3.021 (2.161–4.225)	<0.001

### 2.7 Functional Enrichment and Immune Infiltration Analysis

Using the C2 (c2.cp.kegg.v2022.1.Hs.symbols.gmt) and C5 (c5.go.v2022.1.Hs.symbols.gmt) subsets obtained from the Molecular Signature Database, gene set enrichment analysis was conducted to evaluate possible biological variations between high‐ and low‐risk score clusters, and terms were deemed important if their modified *p* value was below 0.05 and their false discovery rate was below 0.25.

### 2.8 Immune Infiltration Analysis

Then, the infiltration level of immunocytes in the TIME were calculated via three independent algorithms: MCP‐counter, quanTIseq, and CIBERSORTx [29, 30, 31]. Then, the infiltration level of immunocytes in the TIME were calculated via three independent algorithms: MCP‐counter, quanTIseq, and CIBERSORTx [32] (http://www.immport.org) between high‐risk and low‐risk groups were assessed and compared by the R package “GSVA” [33].

### 2.9 Drug Sensitivity Analysis

The R package “OncoPredict” was made with the objective of predicting drug responses in vivo among cancer patients [34]. The methodology employed by OncoPredict involves fitting the gene expression profile of tissues to the half‐maximal inhibitory concentration of cancer cell lines treated with drugs obtained from the Genomics of Drug Sensitivity in Cancer database (https://www.cancerrxgene.org/) and the gene expression profile of cancer cell lines from the Broad Institute Cancer Cell Line Encyclopedia database (https://portals.broadinstitute.org/ccle_legacy/home). The drug sensitivity of 198 drugs was calculated utilizing this approach, and the high‐ and low‐risk groups was explored comparatively.

### 2.10 Quantitative Real‐Time PCR (qRT‐PCR)

To assess the expression patterns of 12 paired samples obtained from patients undergoing fibrobronchoscopic lung biopsy at the Department of Respiratory Diseases, we used qRT‐PCR techniques. Ethical approval for this research was provided by the Ethics Committee of the Second Affiliated Hospital of the Army Medical University. With the SPARKeasy Improved Tissue/Cell RNA Kit, we extracted total RNA from both LUAD tissue and nearby normal tissue samples. This RNA was then converted to complementary DNA by reverse transcription using SPARKscript II All‐in‐one RT SuperMix (Sparkjade, Shandong, China). SYBR Green Master Mix (Sparkjade, Shandong, China) was adopted in the qRT‐PCR process to amplify three key genes. The PCR primers adopted herein are detailed in Supporting Information: Table S2. The relative expression degrees of these hub BMRGs was quantified with the 2^−ΔΔCT^ approach.

## 3 Results

### 3.1 Exploration of BMRGs in LUAD

**Figure 2:**
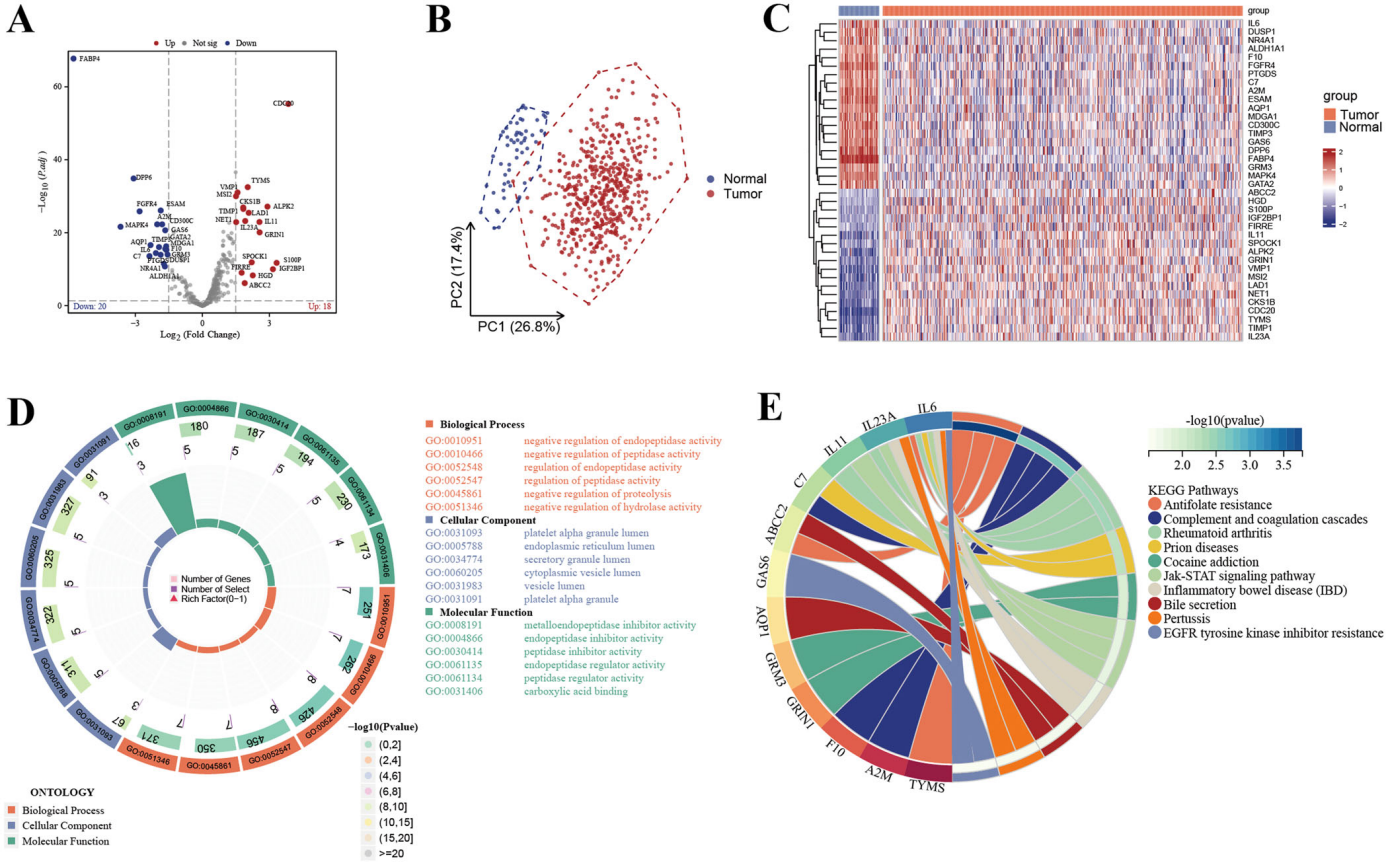
Exploration of differentially expressed butyrate metabolism‐related genes (BMRGs) in lung adenocarcinoma (LUAD). A, Volcano plot for screened BMRGs. B, Principal component analysis for the expression of BMRGs to discriminate tumors from normal samples in TCGA‐LUAD cohort. C, Heatmap for 38 BMRGs in TCGA‐LUAD cohort. D, GO pathway analysis of the 38 BMRGs. E, KEGG pathway analysis of the 38 BMRGs.

**Figure 3:**
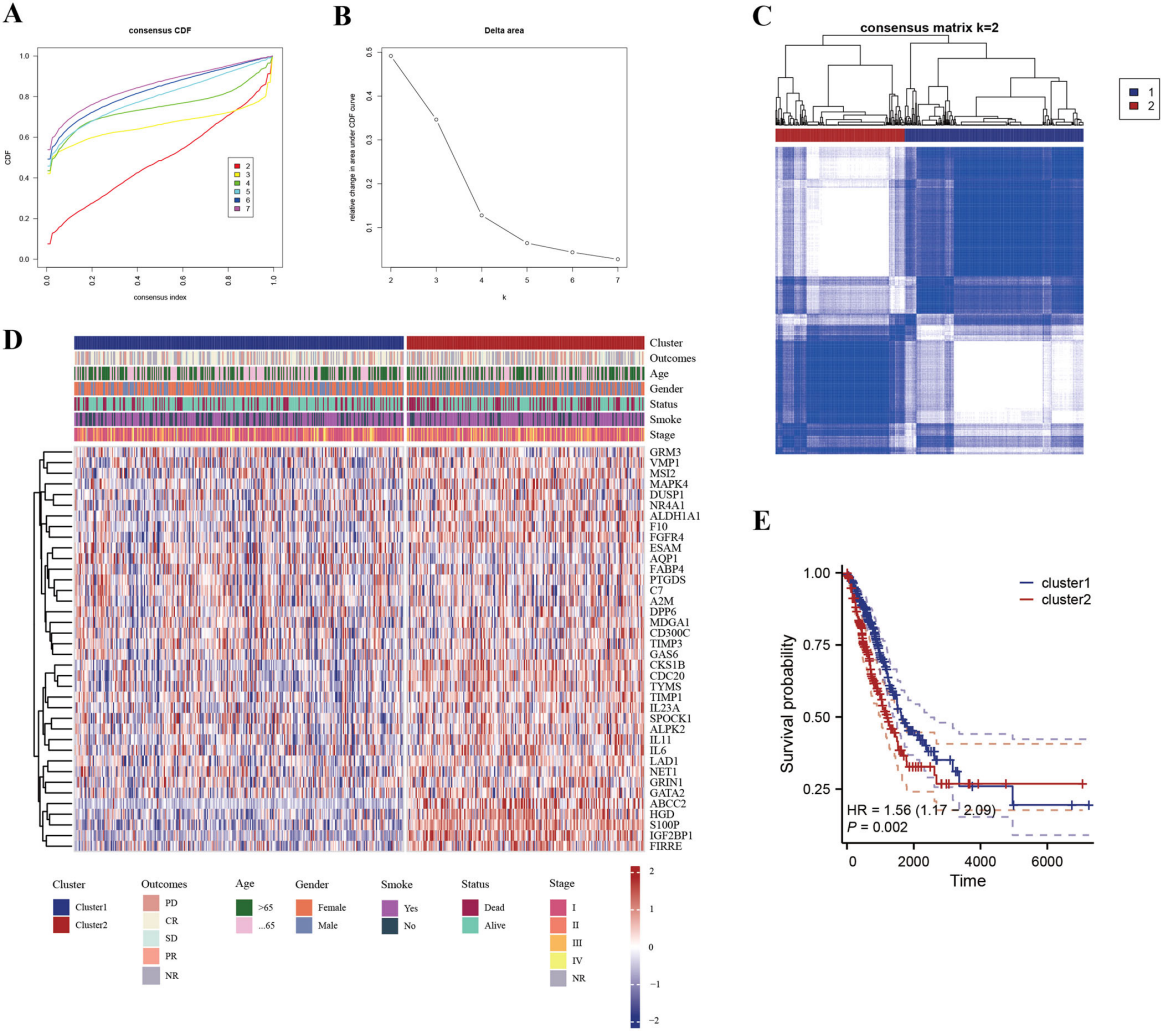
Subtype analysis based on differentially expressed butyrate metabolism‐related genes. A, Consensus cumulative distribution function (CDF) curve plot. B, Consensus clustering CDF for *k* = 2–7. C, Consensus matrix when *k* = 2. D, Heatmap for the relationship of 38 BMRGs and subtype characteristics in LUAD patients. E, Kaplan–Meier curves for the overall survival of LUAD patients in different clusters.

There were 38 differentially expressed BMRGs were identified (Figure 2A), and the PCA results showed that these BMRGs can successfully discriminate LUAD samples from heathy controls (Figure 2B). The expression modes of these BMRGs was visualized with a heatmap (Figure 2C). Furthermore, to investigate the potential functions of these dysregulated BMRGs, GO and KEGG functional enrichment analyses were made, the results indicated that these BMRGs may be involved in many important pathways (Figure 2D,E), such as negative control of endopeptidase activity, platelet alpha granule lumen, metalloendopeptidase inhibitor activity, antifolate resistance, and complement and coagulation cascades. These pathways are relevant because butyrate, as a HDAC inhibitor, influences gene expression involved in immune regulation and enzymatic activity, which plays a critical role in the TIME. For instance, the modulation of complement pathways and peptidase activity may affect immune responses within tumors, reflecting the potential impact of butyrate metabolism on LUAD progression. Further investigation into these enriched pathways will help clarify the precise molecular mechanisms by which butyrate metabolism affects the TIME in LUAD. To further categorize LUAD patients based on their butyrate metabolism profiles, we employed unsupervised consensus clustering using the expression levels of the 38 BMRGs. Our analysis revealed that the pam clustering approach with two clusters (Cluster 1, *n* = 298; Cluster 2, *n* = 215) yielded the most distinct patient subpopulations. Based on the CDF curve, *k* = 2 was determined as the optimal number of clusters (Figure 3A–C). Subsequently, the correlation between BMRGs expression and clinical features (Figure 3D) was dug. Notably, a significant diversity in OS was discovered between the two clusters of LUAD patients: 1.56, 95% confidence interval: 1.17–2.09, log‐rank *p* = 0.002) (Figure 3E).

### 3.2 Model Construction

**Figure 4:**
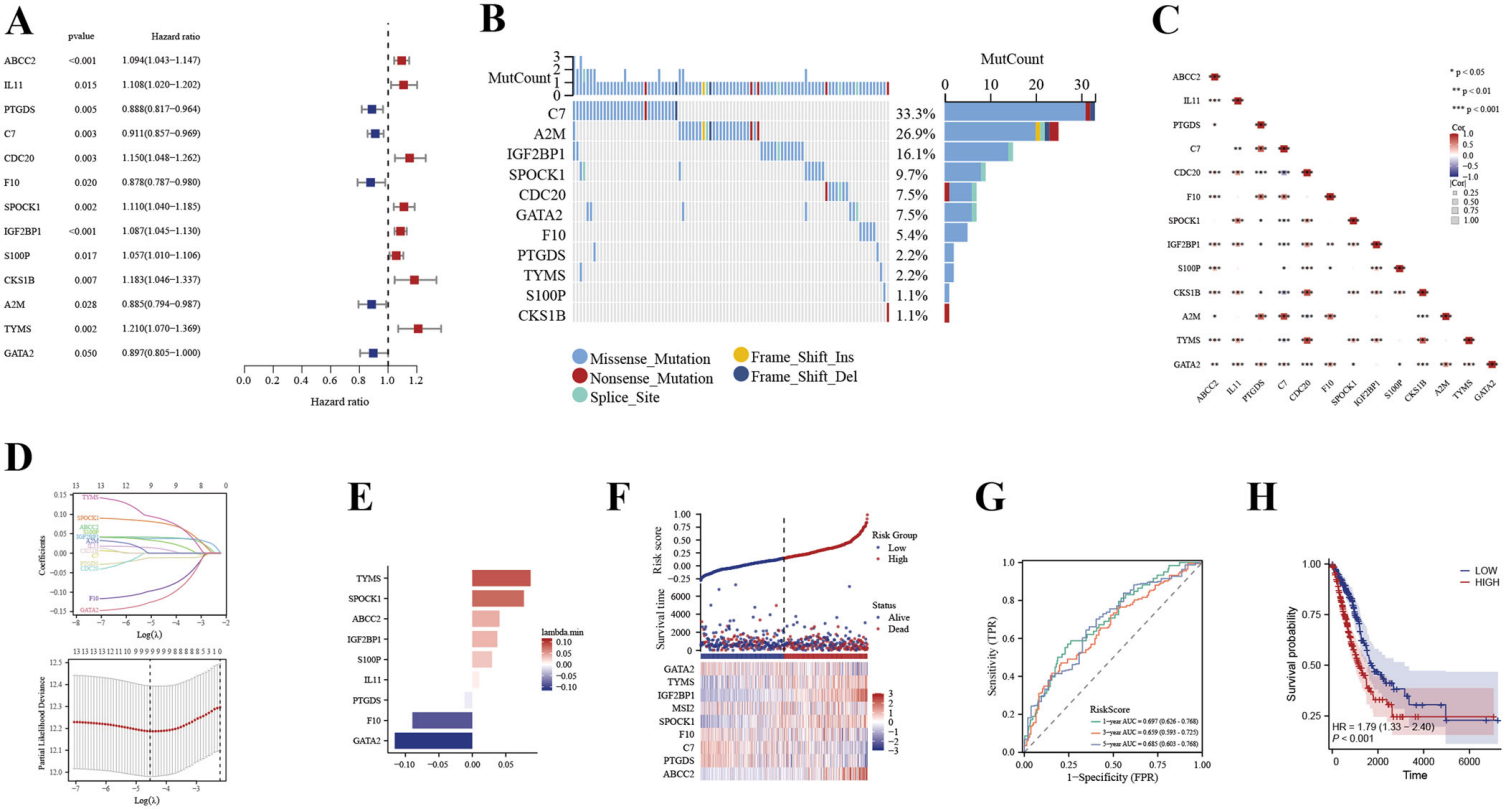
Construction of a prognostic model for LUAD patients based on prognostic butyrate metabolism‐related genes. A, Univariate Cox regression analysis revealed the prognostic BMRG candidates. B, Oncoplot showed the mutation map of BMRG candidates. C, Heatmap for the correlation of the BMRG candidates. D, The LASSO regression algorithm was used to select the optimal variable with a 10‐fold cross‐validation method, and nine hub BMRGs were identified. E, The LASSO coefficient of each hub BMRG. F, The distribution of risk score, survival status, and the expression levels of coefficients in the prognostic signature. G, The time‐dependent ROC curves for the prognostic signature in the TCGA cohort. H Kaplan–Meier curves for the overall survival of LUAD patients in high‐ and low‐risk score groups.

The TCGA‐LUAD cohort acted as a training dataset to delve into the prognostic importance of 38 BMRGs. To identify those BMRGs linked to patient survival, a univariate Cox analysis was conducted, ultimately selecting 13 genes, these 13 genes are presented in Figure 4B, with C7 and A2M genes exhibiting the highest mutation frequencies. Subsequently, an analysis of the correlation among these 13 prognosis‐related genes revealed that CDC20‐TYMS was the most positively correlated gene pair, whereas CDC20‐C7 was the most negatively correlated pair (Figure 4C). To further refine our analysis, the LASSO logistic regression algorithm was adopted to pinpoint the hub BMRGs from the pool of 13. Using the LASSO Cox regression analysis, we zeroed down on nine genes crucial for establishing fatty acid risk score models: TYMS, SPOCK1, ABCC2, IGF2BP1, S100P, IL11, PTGDS, F10, and GATA2 (Figure 4D). The corresponding coefficients of each hub BMRG in the prognostic signature were exhibited (Figure 4E). Notably, the high‐risk group displayed greatly higher ratio of deceased LUAD patients by comparing with the low‐risk group (Figure 4F). For evaluating the prediction of our prognostic signature, we plotted and compared time‐dependent ROC and Kaplan–Meier curves. The results revealed that the TCGA cohort's 1‐, 3‐, and 5‐year OS areas under the ROC curves (AUCs) were 0.697, 0.659, and 0.685 (Figure 4G). In addition, the external independent dataset GSE72094 yielded similar results, with the 1‐, 3‐, and 5‐year OS AUCs being 0.709, 0.675, and 0.764, respectively (Supporting Information: Figure S2). Furthermore, Kaplan–Meier analysis demonstrated that LUAD patients categorized as low‐risk had a greatly longer OS by comparing with their high‐risk counterparts (Figure 4H). Consistent trends were observed in the external validation cohorts (Supporting Information: Figure S1).

### 3.3 Validation of the Hub BMRGs

**Figure 5:**
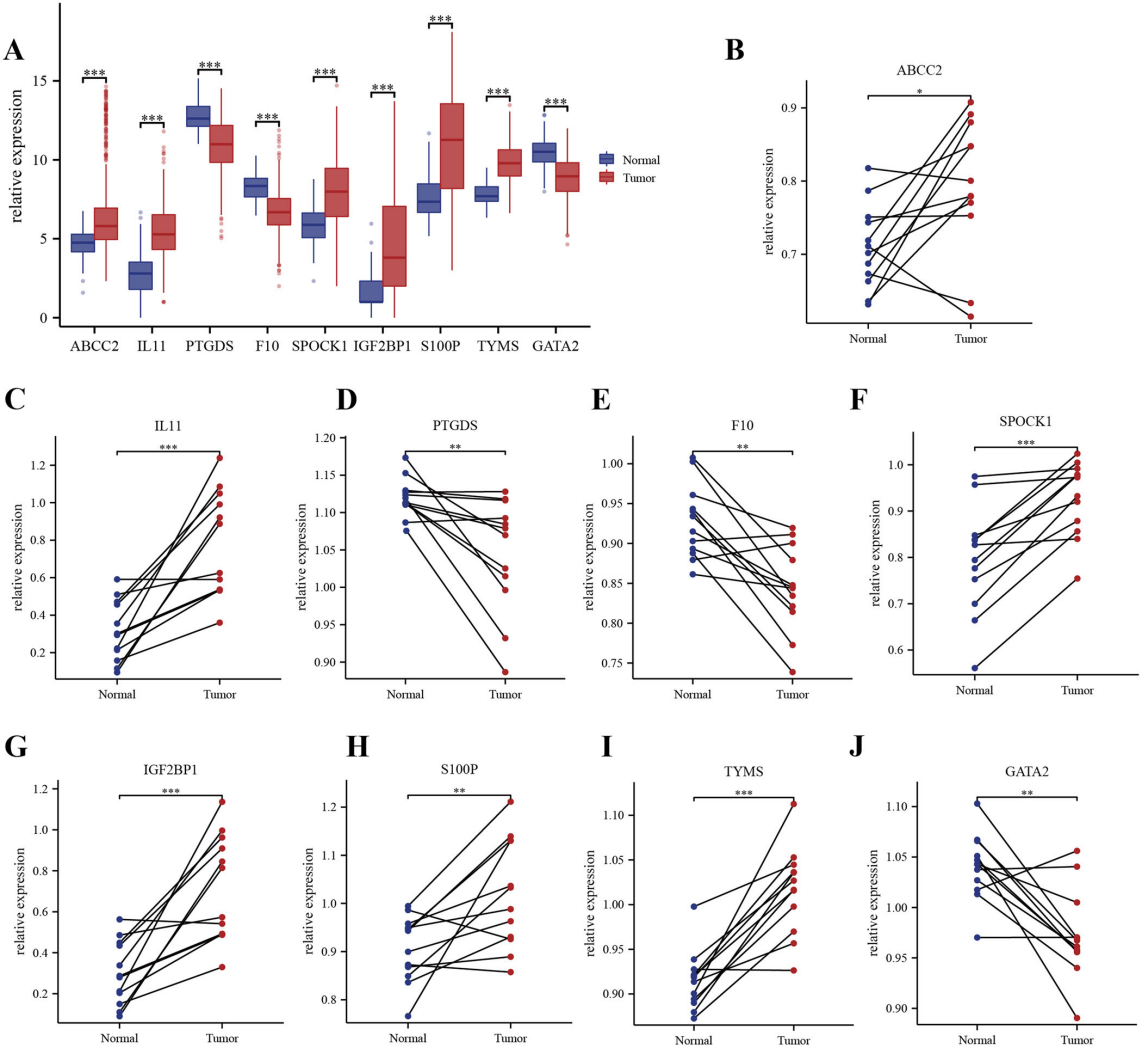
Experiment validation of hub butyrate metabolism‐related genes (BMRGs) in human samples. A, The expression of hub BMRGs in TCGA cohort. B–J, The mRNA levels of hub BMRGs (ABCC2, IL11, PTGDS, F10, SPOCK1, IGF2BP1, S100P, TYMS, and GATA2) in 12 pairs of LUAD and their paired adjacent normal tissues were measured by qRT‐PCR (paired *t*‐test, ∗*p* < 0.05; ∗∗*p* < 0.01; ∗∗∗*p* < 0.001).

The expression degrees of hub BMRGs in TCGA cohort were exhibited (Figure 5A). The qRT‐PCR was performed on 12 paired LUAD samples to investigate the expression degrees of various genes in cancerous and normal lung tissues. According to the outcomes, the expression of GATA2, F10, and PTGDS was greatly lower in cancerous tissues than in normal lung tissues. Conversely, it was discovered that the expression of TYMS, IL11, SPOCK1, S100P, IGF2BP1, and ABCC2 is greatly higher in LUAD tissues (Figure 5B–J, *p* < 0.05), which were consistent with the bioinformatics.

### 3.4 Quantifying the Risk of Individual LUAD Patients

**Figure 6:**
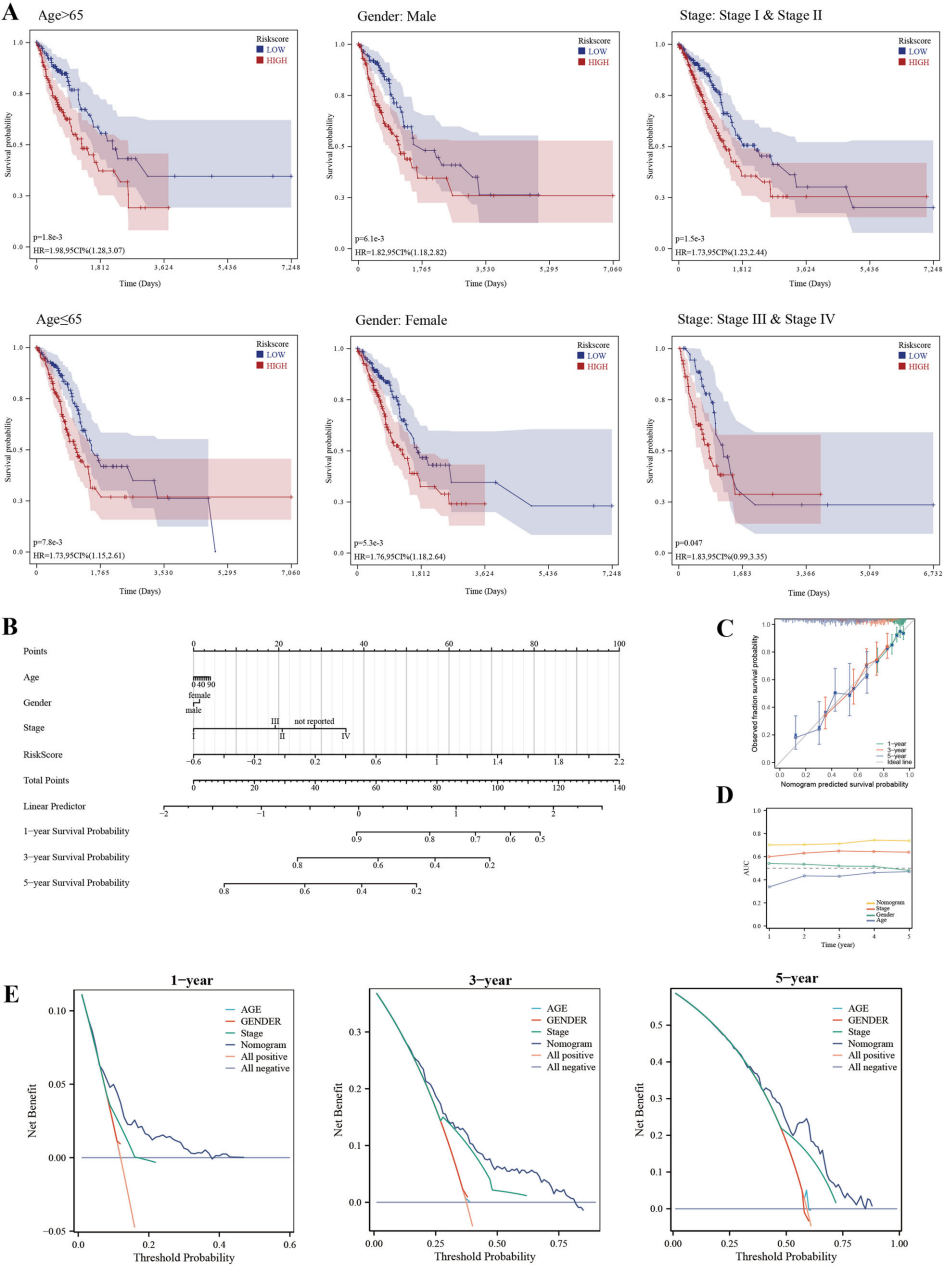
Quantifying individual butyrate metabolism‐related risks. A, Subgroup analysis of risk in patients with different clinical characteristics. B, Butyrate metabolism‐related risk scores‐based nomograms were used to quantify individual patient score. C, Nomogram calibration curves at 1, 3 and 5 years. D, Scores of area under curve of nomogram. E, Nomogram DCA curve in 1, 3 and 5 years.

We assessed the variations in risk scores across various subgroups based on age, gender, and stage to ascertain the association between clinical features and risk score (Figure 6A). Nomograms were established to better calculate the risk in each individual LUAD patient (Figure 6B). Nomogram model showed satisfied accuracy and stability in 1, 3, and 5 years, according to the nomogram correction curve (Figure 6C). According to the results obtained from time‐dependent ROC analysis, the nomogram model exhibited the highest predictive accuracy in comparison to the clinical characteristics, as demonstrated by the greater area under the curve (Figure 6D). DCA was then used to determine the nomogram model's benefits for decision‐making. The findings demonstrated that the nomogram was appropriate for assessing the risk of LUAD patients over 1, 3, and 5 years (Figure 6E).

### 3.5 Functional Enrichment Analysis

**Figure 7:**
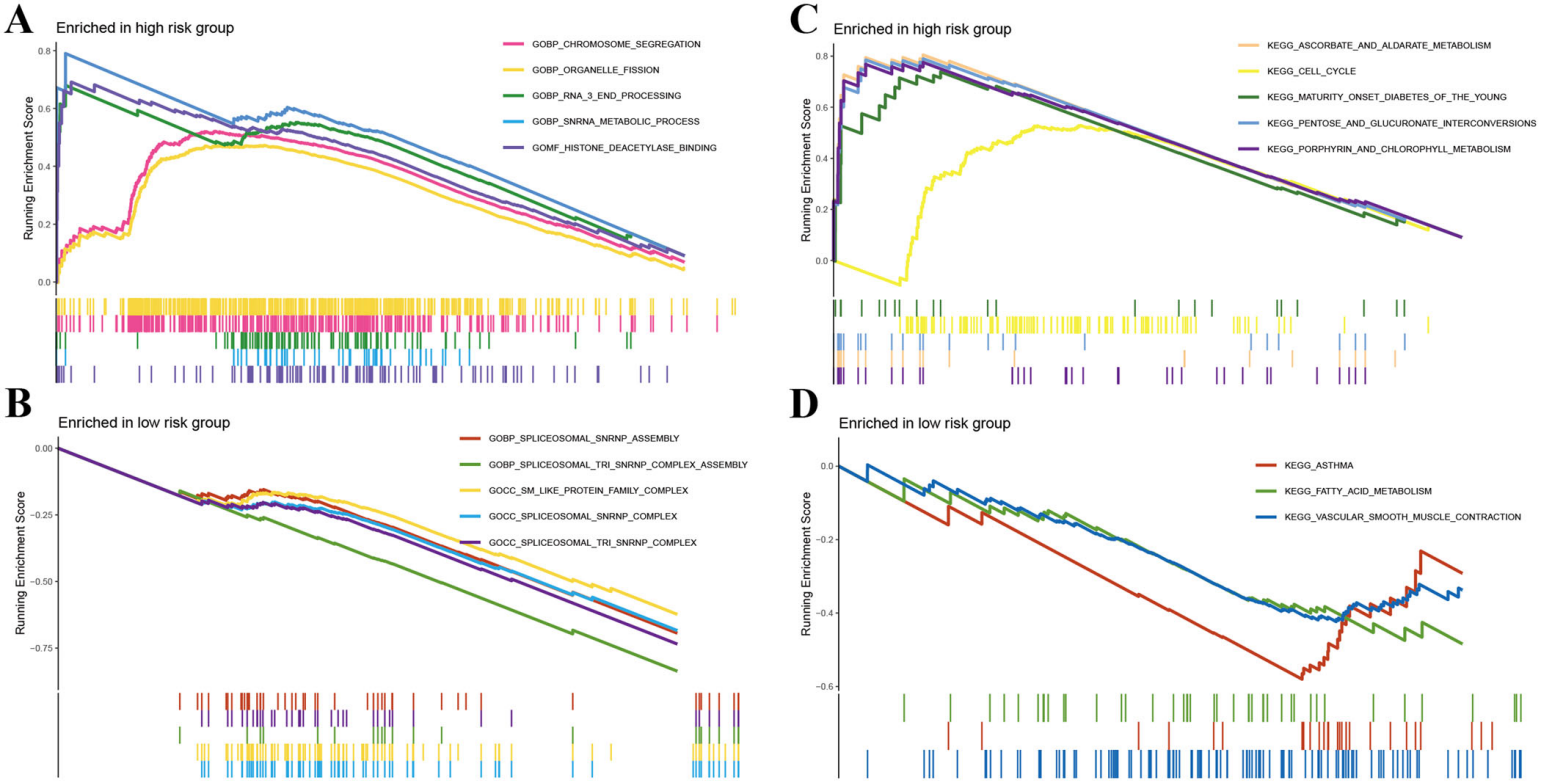
Gene Sets Enrichment Analysis of different butyrate metabolism‐related risk subgroups. A, Gene Sets Enrichment Analysis (GSEA) of GO pathways in the butyrate metabolism‐related high‐risk subgroups. B, GSEA of GO pathways in the butyrate metabolism‐related low‐risk subgroups. C, GSEA of KEGG pathways in the butyrate metabolism‐related high‐risk subgroups. D, GSEA of KEGG pathways in the butyrate metabolism‐related low‐risk subgroups.

GSEA was made to examine the underlying processes of the survival difference. On basis of the GO enrichment analysis, the main enrichment of high‐risk group was made in the chromosome segregation and RNA‐related processes (Figure 7A). In the low‐risk group, spliceosomal‐related processes were mostly observed (Figure 7B). According to the KEGG outcomes, the enrichment of the high‐risk group was made in the ascorbate and aldarate metabolism, cell cycle, and pentose and glucuronate interconversions (Figure 7C), while the low‐risk group displayed enrichment mostly in the fatty acid metabolism, asthma, and vascular smooth muscle contraction (Figure 7D).

### 3.6 Various Immunological Characteristics Were Discoverd in the High‐Risk and Low‐Risk Groups

**Figure 8:**
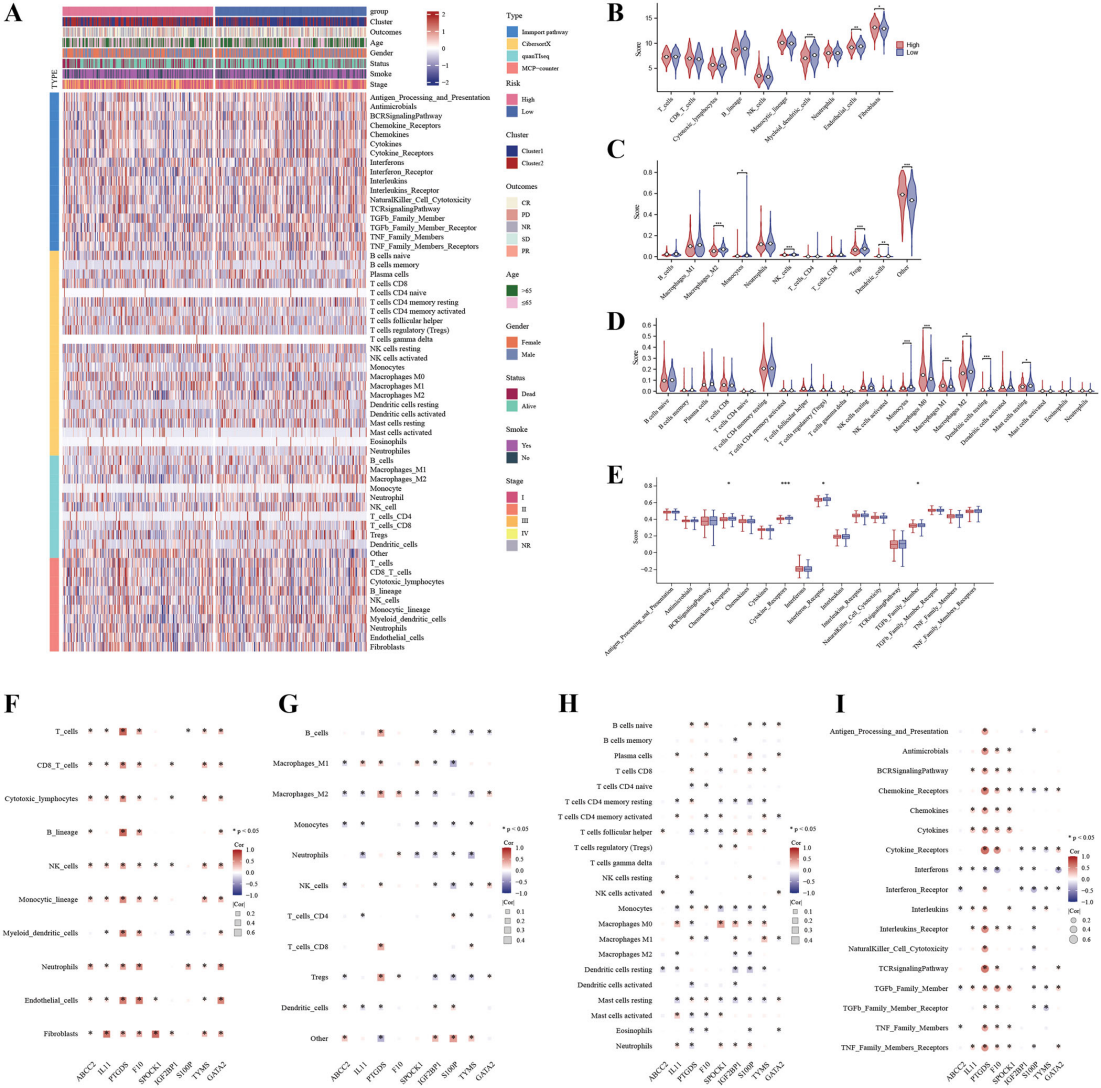
The immune infiltration characteristics in different butyrate metabolism‐related risk subgroups. A, The heatmap of immune cells and immune pathway activities. Distinct immune cell infiltration scores of butyrate metabolism‐related risk subgroups using. B, MCP‐counter, C, quanTIseq, and D, CIBERSORTx algorithms. E, Distinct immune pathway activities of butyrate metabolism‐related risk subgroups. F–I, The correlation heatmap of hub butyrate metabolism‐related genes and immune infiltration characteristics (∗*p* < 0.05; ∗∗*p* < 0.01; ∗∗∗*p* < 0.001).

This research sought to explore the immunological features of high‐ and low‐risk groups in LUAD patients. To achieve this, we evaluated immune pathway activity and immunocyte infiltration levels (Figure 8A). Specifically, we found that myeloid dendritic cells, regulatory T cells, and macrophage infiltration levels varied greatly between the high‐ and low‐risk groups (Figure 8B–D). Moreover, the activity of cytokine receptor‐related pathways was greatly lower in the high‐risk group by comparing with the low‐risk group (Figure 8E). Furthermore, we assessed the association coefficients between the hub BMRGs and immune characteristics in LUAD (Figure 8F–I). Notably, our results demonstrated that PTGDS was strongly correlated with T cells, chemokine‐related pathways, and the T‐cell receptor (TCR) signaling pathway. These outcomes suggest that PTGDS may exert a key effect on modulating the immune reaction in LUAD, potentially implicating its relevance as a therapeutic target.

### 3.7 Anticancer Drug Sensitivity Analysis

**Figure 9:**
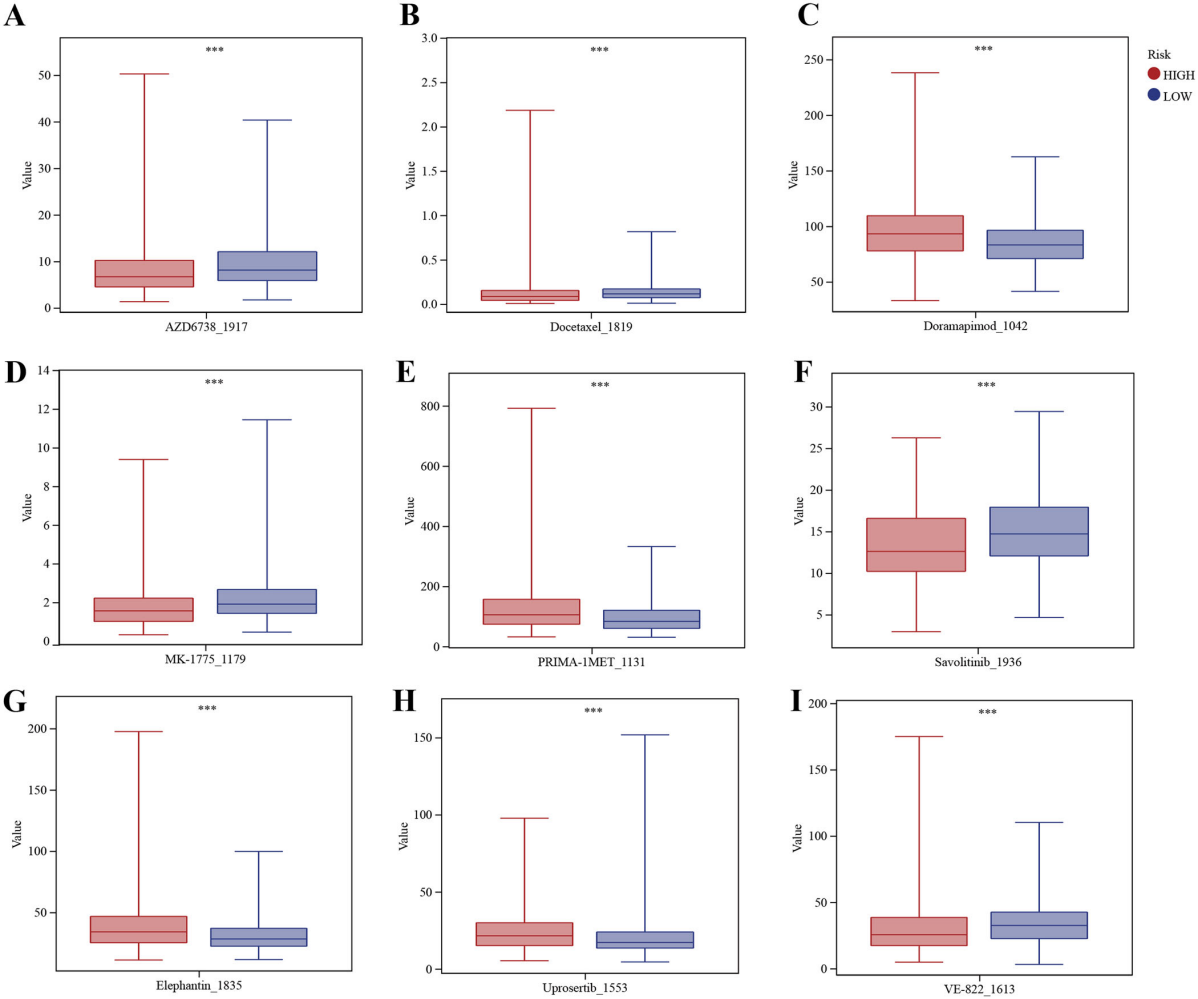
Butyrate metabolism‐based signature is associated with chemotherapy response. A–I, Predicted sensitivity scores of Doramapimod_1042, PRIMA‐1MET_1131, Elephantin_1835, and Uprosertib_1553, which were candidate chemotherapeutic agents for high butyrate metabolism‐related risk score patients; Predicted sensitivity scores of AZD6738_1917, Docetaxel_1819, MK‐1775_1179, Savolitinib_1936, and VE‐822_1613, which were candidate chemotherapeutic agents for low butyrate metabolism‐related risk score patients (∗∗∗*p* < 0.001).

A total of 198 responses to anticancer drugs were assessed and compared between two risk groups. Among these, nine drugs demonstrated statistically significant differences (with FDR < 0.01) between the groups, as illustrated in Figure 9A–I. Specifically, patients in the high‐risk group displayed higher sensitivity to four drugs, namely Doramapimod_1042, PRIMA‐1MET_1131, Elephantin_1835, and Uprosertib_1553, while patients in the low‐risk group showed higher sensitivity to five medicine, such as AZD6738_1917, Docetaxel_1819, MK‐1775_1179, Savolitinib_1936, and VE‐822_1613. This method represents a powerful tool for predicting drug responses in cancer patients, as it integrates information from large‐scale drug sensitivity databases with gene expression data from patient tissues. The results obtained from this approach may facilitate the selection of optimal treatment strategies for individual cancer patients, thereby improving the efficacy of cancer therapy.

## 4 Discussion

By modifying the expression of genes, butyrate, a naturally occurring HDAC inhibitor, has the power to suppress proliferation, stimulate apoptosis, and regulate the cell cycle in cancer cells [35, 36]. Butyrate performs a variety of activities in malignancies, which suggests a very intricate regulatory system. It is vital to thoroughly investigate the function of butyrate to illustrate the probable regulating system of butyrate metabolism in TIME. Research on the metabolism of butyrate in LUAD TIME is limited thus far.

A total of 38 BMRGs were dysregulated, which can well discriminate tumor tissues from normal tissues. The functional enrichment analysis suggested these genes are closely associated with enzymatic activity and immunity, prompting that butyrate metabolism may be crucial in LUAD. Afterwards, utilizing bioinformatics and statistical techniques, we thoroughly evaluated the prognostic prediction accuracy of BMRGs in LUAD, and nine genes (TYMS, SPOCK1, ABCC2, IGF2BP1, S100P, IL11, PTGDS, F10, and GATA2) were screened out from 38 BMRGs to establish the prognosis model and generate the risk score. The model's performance is nearly consistent with Yang's [37]. However, butyrate‐related biomarkers, known for their role in modulating immune responses, anti‐inflammatory effects, and maintaining epithelial integrity, offer additional biological insights. These properties suggest that the inclusion of butyrate‐related markers could enhance the model's capability to capture subtle biological variations associated with disease progression, making it a valuable addition for personalized prognosis. Moreover, our study findings demonstrate that the developed prognosis model exhibited superior predictive performance in early‐stage LUAD patients. Notably, the high‐risk score appeared as an important and independent negative prognostic index for totall survival in LUAD patients. The patients in the study range in age from 33 to 88 years old. Survival differences between younger and older age groups are notable. Patients over 65 years of age in the high‐risk group show significantly lower survival rates compared to those in the low‐risk group, whereas younger patients (≤65) also experience survival differences between the high and low‐risk groups, though these differences are less pronounced in younger individuals. The majority of the patients in this study are white, which adds some limitations regarding the generalizability of the findings across different ethnic groups. Besides, a nomogram incorporating both prognosis related BMRGs and clinical characteristics that can effectively predict the survival outcomes in LUAD patients was developed and verified. Specifically, the nomogram can be used alongside routine clinical assessments to offer personalized survival predictions, helping clinicians stratify patients by risk and tailor treatment strategies accordingly. It can be incorporated into electronic health record (EHR) systems to facilitate real‐time usage, allowing for quick visualization of patient prognosis during consultations. Additionally, integrating the nomogram with other clinical decision‐making tools, such as multidisciplinary team discussions, will further enhance its utility. Training sessions for healthcare providers and the development of user‐friendly software interfaces will also ensure smooth adoption in clinical practice. Remarkably, our results indicate that butyrate metabolism‐based features hold exceptional accuracy and could surpass traditional clinicopathological characteristics in predicting the prognosis of LUAD.

This investigation showcases the impact of BMRGs on LUAD TIME. By assessing risk scores in both high‐risk and low‐risk LUAD patients, differential levels of immunocyte infiltration and immune pathway activities were detected, suggesting an association between the prognostic hub BMRGs and these immune characteristics. These findings highlight the relevance of BMRGs in the modulation of LUAD TIME. According to our analysis results, the hub prognosis BMRG PTGDS is strongly correlated with T cells, chemokine‐related pathway, and TCR signaling pathway. PTGDS is a glutathione‐independent prostaglandin D synthase with the ability to catalyzes prostaglandin 2 production and transports lipophilic substances [38]. According to recent studies, PTGDS was found to be overexpressed in hepatocellular adenoma [39], ovarian carcinoma [40], and malignant melanomas [41]. Other researchers, however, discovered that PTGDS was downregulated and prevented tumor growth in gastric cancer [42], cervical squamous cell carcinoma [43], lung tumors [44], and prostate tumors [45]. More importantly, the expression status of PTGDS was reported correlated with T cells, which is consistent to our findings. The interaction between butyrate metabolism and T cells was widely reported: Butyrate has been shown to promote the development and function of Tregs [46]. Tregs are a type of T cell that helps to maintain immune homeostasis by suppressing the activity of other T cells that can lead to excessive inflammation and tissue damage [47, 48, 49]. Research has also shown that butyrate can influence the differentiation and function of other types of T cells. For example, butyrate has been shown to promote the differentiation of T helper 17 (Th17) cells, which take part in immune responses. Butyrate can also influence the activity of cytotoxic T cells, which exert a key effect on the immune response against cancer [10, 50]. Studies have also shown that butyrate can enhance the anti‐tumor activity of cytotoxic T cells, indicating therapeutic potential in the treatment of cancer [51]. Overall, the relationship between T cells and butyrate is complex, with butyrate having both beneficial and potentially detrimental effects on T cell function relying on the specific T cell subtype and context. However, the underlying interaction between PTGDS and TIME in LUAD has not yet been reported, which could be a promising new approach for future studies. Last, we performed OncoPredict and found the distinct sensitivities of nine drugs in high‐ and low‐risk group, which may advance the development of customized medicine in the field of oncology, this approach could contribute to more precise and effective cancer treatment, and ultimately, improve OS rates for patients with LUAD.

The current research made a systematic analysis of butyrate metabolism in LUAD, leading to the generation of valuable insights and information. However, further comprehensive investigation is needed to elucidate the regulatory mechanisms underlying butyrate metabolism in LUAD.

The current study has several limitations. Clinical factors such as age, gender, smoking status, cancer stage, and prior treatments could act as confounders in the relationship between BMRGs and patient outcomes. Although the study accounts for some variables, including age, gender, and cancer stage, further adjustments for smoking status, comorbidities, and other clinical factors could enhance the robustness of the results. Genomic heterogeneity, including variations in genetic backgrounds or mutations unrelated to butyrate metabolism, may also affect the findings. A more detailed discussion of the genomic variability in LUAD and its interaction with BMRGs would provide deeper insights. Additionally, variability in the TIME, such as differences in immune cell infiltration and cytokine profiles among patients, could introduce confounding effects. Addressing how these immune‐related factors were managed would improve the study's reliability. Furthermore, while external datasets, such as GEO datasets, were used for validation, it is essential to assess whether these datasets share similar distributions of confounders. Any discrepancies between datasets could limit the generalizability of the findings, and acknowledging such differences would strengthen the interpretation of the results.

## 5 Conclusions

A prognostic signature encompassing genes related to butyrate metabolism was developed and subsequently assessed for its impact on LUAD progression and prognosis. The findings yielded novel prognostic markers and potential targets for personalized intervention in LUAD. Future research should explore the mechanistic role of identified genes in butyrate metabolism and their impact on LUAD progression and the TIME. Experimental validations, such as functional studies and in vivo models, are needed to confirm the associations and support clinical translation. Investigating the interaction between PTGDS, T cells, and chemokine‐related pathways could uncover new therapeutic strategies and potential targets for personalized treatments.

## Author Contributions

All the authors participated in the present study, including conception and design (FY, XY, WQ, and ZJ), data collection, data analysis, drafting or critically revising the article (ZJ, WJ, WXY, and YYT), as well as study supervision (FY and ZJ). All the authors have read and approved the final version submitted.

## Ethics Statement

The authors are accountable for all aspects of the work in ensuring that questions related to the accuracy or integrity of any part of the work are appropriately investigated and resolved. The study was conducted in accordance with the Declaration of Helsinki (as revised in 2013). This study was reviewed and approved by the Ethics Committee of Second Affiliated Hospital of Army Medical University (2023‐NO.076‐02). All patients signed a general informed consent of their anonymized clinical data.

## Conflicts of Interest

The authors declare no conflicts of interest.

## Supporting information

Supporting information.
