# Supplementary material for: Butyrate Metabolism‐Related Gene Signature in Tumor Immune Microenvironment in Lung Adenocarcinoma: A Comprehensive Bioinformatics Study
Source: Immun Inflamm Dis. 2024 Dec 6;12(12):e70087. doi: 10.1002/iid3.70087 (PMC11621860; doi:10.1002/iid3.70087)
Supplement: Supplementary file 1 — Supporting information. [file IID3-12-e70087-s001.docx]

| **Supplementary table 1: butyrate-metabolism-related genes** |
| --- |
| A2M |
| ABCC2 |
| ACP2 |
| ADH6 |
| AFF1 |
| ALAD |
| ALDH1A1 |
| ALPK2 |
| AQP1 |
| ATP7B |
| C7 |
| CAPN7 |
| CD300C |
| CDH11 |
| CDK4 |
| CKS1B |
| CLCN3 |
| COL6A3 |
| CSF1 |
| CUL1 |
| CYP4A11 |
| DPP6 |
| EIF4G2 |
| ELANE |
| ELK3 |
| EMP3 |
| ERCC2 |
| F10 |
| FES |
| FSTL1 |
| G6PC1 |
| GAA |
| GMPS |
| GRM3 |
| HMGB1 |
| HSPG2 |
| IL11 |
| IL6 |
| IRF2 |
| MPP3 |
| MYL4 |
| NAMPT |
| NCL |
| NF2 |
| NUCB1 |
| PFKL |
| PRKAR2A |
| PYGL |
| RNF141 |
| STAT6 |
| SULT1E1 |
| THBS4 |
| THRA |
| THSD7A |
| TIMP3 |
| TUBA4A |
| TXK |
| USP7 |
| VEGFB |
| ZNF137P |
| CDC20 |
| CDK10 |
| CDKN1A |
| CXCR4 |
| DDR1 |
| EPHB3 |
| GADD45A |
| GATA2 |
| GSTT1 |
| HSPA1A |
| HSPB1 |
| ICAM1 |
| MAPRE1 |
| NET1 |
| NR4A1 |
| POR |
| PRDX1 |
| PRKCD |
| RHOA |
| RNH1 |
| TOB1 |
| ATP5MC3 |
| CD82 |
| CETN2 |
| CLU |
| COX6A1 |
| ESYT2 |
| FABP4 |
| FURIN |
| GDI1 |
| GPRC5A |
| GSTP1 |
| HADHB |
| IFI30 |
| IGF2 |
| MT2A |
| NME2 |
| PCMT1 |
| POLR2L |
| PRKCB |
| PSAP |
| RAB11A |
| S100P |
| SAT1 |
| SEC61B |
| SERPINB2 |
| SPOCK1 |
| TIMP1 |
| UQCRQ |
| AAMDC |
| ABCD3 |
| ACAP3 |
| ADGRB2 |
| ADSS2 |
| AFTPH |
| ARHGEF10 |
| BCL2L1 |
| CALCOCO1 |
| CCN2 |
| CDKN1C |
| CHI3L1 |
| CHMP4B |
| CNKSR1 |
| CORO7 |
| CTNND1 |
| CYB5B |
| CYP1B1 |
| DGKQ |
| DLX4 |
| DNAH17 |
| DSE |
| DUSP1 |
| ENKD1 |
| ESAM |
| FBH1 |
| FNBP4 |
| GAK |
| GALT |
| GGT7 |
| GLI3 |
| GNPTG |
| H1-2 |
| HGD |
| ID2 |
| ID3 |
| ISG15 |
| MBD4 |
| MCAM |
| MIR22HG |
| MYO9B |
| NAALADL1 |
| NCOA1 |
| NOS3 |
| ODF2 |
| PDPK1 |
| PPP1R12C |
| RBMS2 |
| RETSAT |
| RINT1 |
| RMC1 |
| SBNO1 |
| SLC25A16 |
| SLC2A2 |
| SMARCD3 |
| SYNJ1 |
| TGM2 |
| TUSC2 |
| UBR4 |
| ZFP36L2 |
| BDP1 |
| CERS2 |
| COL4A2 |
| DCT |
| DEGS1 |
| FAM193A |
| FGFR4 |
| FIRRE |
| GABPA |
| GAS6 |
| GIT1 |
| GLMN |
| GTF2IRD1 |
| IGF2BP1 |
| INO80D |
| INPP5D |
| KANK1 |
| KLHL42 |
| LINC01004 |
| LUC7L2 |
| MET |
| MPDU1 |
| MTCH2 |
| MTR |
| NRAP |
| OGDH |
| OTUD4 |
| PAF1 |
| PAIP2B |
| PAN3 |
| PCGF3-AS1 |
| RBAK |
| RNF138 |
| SCCPDH |
| ST3GAL4 |
| TCF3 |
| TRIM47 |
| TSEN2 |
| UQCRFS1 |
| USF2 |
| ZFX |
| ZNF449 |
| FOS |
| GRN |
| MT1E |
| MT1H |
| MYH9 |
| PLCB4 |
| TMSB4X |
| ATP5F1D |
| MAP1LC3B |
| NEU1 |
| NPC2 |
| PER1 |
| UBE2V1 |
| VMP1 |
| ZFP36 |
| A1CF |
| CALML4 |
| CREM |
| HNMT |
| MTDH |
| PI4KAP1 |
| PPIL6 |
| SOS2 |
| TBC1D23 |
| ZNF148 |
| CACNA2D4 |
| CKAP2 |
| CSDE1 |
| FAIM |
| FAM136A |
| IL23A |
| MYB |
| NUCKS1 |
| PHF10 |
| SRSF8 |
| SYK |
| TYMS |
| UBTF |
| ZNF367 |
| ANP32A |
| ARID1B |
| ATP5F1B |
| CDK5RAP2 |
| CDK6 |
| CHDH |
| CTDSP1 |
| DANCR |
| DHX9 |
| EIF4A1 |
| ETS2 |
| GRIN1 |
| HMGB2 |
| HNRNPH1 |
| IFITM2 |
| INPPL1 |
| ITPR2 |
| KHDRBS1 |
| KMT5B |
| MAPK4 |
| MAZ |
| MBNL3 |
| MDGA1 |
| MGME1 |
| MLXIP |
| MSI2 |
| NAP1L1 |
| NCBP3 |
| NSD2 |
| PCNA |
| PLCB2 |
| RFX7 |
| RNF180 |
| RPRD2 |
| SCAF11 |
| SESTD1 |
| SLC45A1 |
| SMC2 |
| SORBS2 |
| ST18 |
| STAT2 |
| TIA1 |
| TMPO |
| TOP2B |
| TUBB4B |
| ZNF529 |
| ACADS |
| ADK |
| AGO2 |
| ATF4 |
| BCL2L12 |
| BTF3 |
| DNAJA4 |
| FUS |
| IFIT1 |
| INPP5A |
| KCNS2 |
| LAD1 |
| MBTD1 |
| ME1 |
| MLH1 |
| PCBP2 |
| PSMB5 |
| SMPD1 |
| STC2 |
| TARS1 |
| XBP1 |
| XPOT |
| ADIRF |
| BGN |
| DDX21 |
| DRAM2 |
| KAT7 |
| LARP1B |
| NCKAP1 |
| NFKB2 |
| PCBP1 |
| PPP1R8 |
| PTGDS |
| RPL41 |
| TP53 |
| AKT1 |
| DCP1A |
| DCP2 |
| DIS3 |
| EXOSC1 |
| EXOSC2 |
| EXOSC3 |
| EXOSC4 |
| EXOSC5 |
| EXOSC6 |
| EXOSC7 |
| EXOSC8 |
| EXOSC9 |
| MAPKAPK2 |
| XRN1 |
| YWHAB |
| ZFP36L1 |

| **Supplementary table 2: The PCR primers** | | |
| --- | --- | --- |
| Gene symbol | Forward Primer | Reverse Primer |
| *TYMS* | CTGCTGACAACCAAACGTGTG | GCATCCCAGATTTTCACTCCCTT |
| *IL11* | CGAGCGGACCTACTGTCCTA | GCCCAGTCAAGTGTCAGGTG |
| *GATA2* | GCAACCCCTACTATGCCAACC | CAGTGGCGTCTTGGAGAAG |
| *F10* | AACCCCAAGGCCGAATTGT | CGCGATCAGGTTCCTCCAG |
| *PTGDS* | GGCGTTGTCCATGTGCAAG | GGACTCCGGTAGCTGTAGGA |
| *SPOCK1* | CCCAACCACGGCAATTTCCTA | ATCGTCTCGAAAGCGGTTCC |
| *S100P* | AAGGATGCCGTGGATAAATTGC | ACACGATGAACTCACTGAAGTC |
| *IGF2BP1* | GCGGCCAGTTCTTGGTCAA | TTGGGCACCGAATGTTCAATC |
| *ABCC2* | CCCTGCTGTTCGATATACCAATC | TCGAGAGAATCCAGAATAGGGAC |
| *GAPDH* | ATCAAGAAGGTGGTGAAGCAGG | CGTCAAAGGTGGAGGAGTGG |


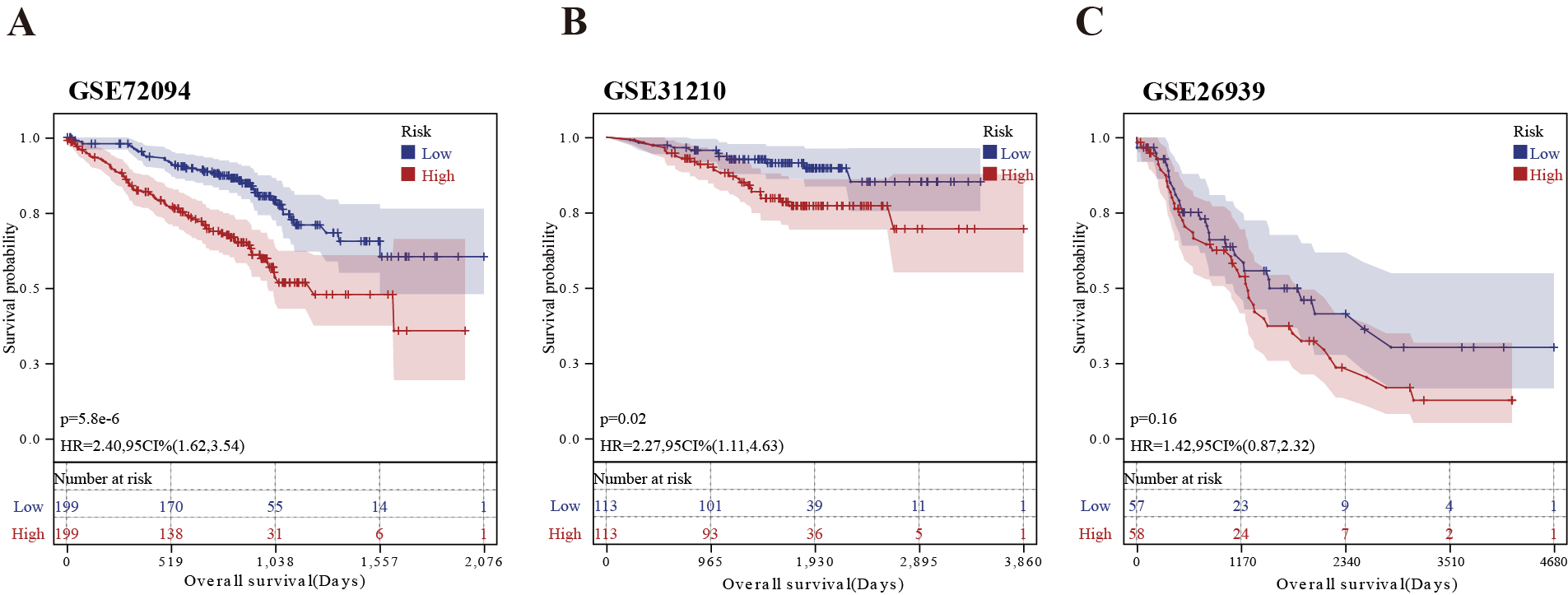


Figure S1. External validation of butyrate metabolism-related risk scores in GEO cohorts. Kaplan–Meier curves for the overall survival of LUAD patients in high- and low-risk score groups in (A) GSE72094, (B) GSE31210, and (C)GSE26939 cohorts.


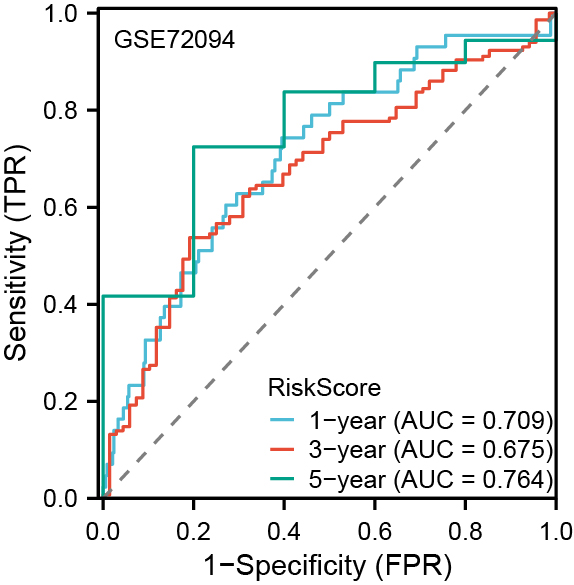


Figure S2. External validation of the the time-dependent ROC curves for the prognostic signature in the GSE72094 cohort.
